# NLP6/7‐mediated nitrate signaling facilitates high‐affinity potassium uptake and enhances tolerance to low potassium environments in Arabidopsis

**DOI:** 10.1111/tpj.71172

**Published:** 2026-10-14

**Authors:** Kosuke Usuda, Mailun Yang, Namie Ohtsuki, Nana Sugimura, Daiki Teshima, Yasuhito Sakuraba, Shuichi Yanagisawa

**Affiliations:** ^1^ Plant Functional Biotechnology, Agro‐Biotechnology Research Center, Graduate School of Agricultural and Life Sciences The University of Tokyo Yayoi 1‐1‐1, Bunkyo‐ku Tokyo 113‐8657 Japan

**Keywords:** potassium acquisition, potassium deficiency, nitrate, NLP, nutrient signaling, Arabidopsis

## Abstract

Balancing the acquisition of the macronutrients nitrogen (N) and potassium (K) is critical for plant growth, yet the mechanisms that balance N and K acquisition remain poorly understood. Here, we show that the NIN‐LIKE PROTEIN7 (NLP7) transcription factor, which mediates nitrate signaling, plays a crucial role in promoting K acquisition in Arabidopsis. K^+^ uptake activity was reduced in the *nlp7* mutant and *nlp6nlp7* double mutant but enhanced in the *NLP7*‐overexpressing line. Consistently, these mutants were hypersensitive to K deficiency, whereas the *NLP7*‐overexpressing line displayed increased tolerance. Transcriptome analysis of the *nlp6nlp7* mutant suggested that NLP7 activates several critical K‐related genes, including *HAK5*, a transporter gene essential for K^+^ uptake from soil, and *SKOR*, a channel gene critical for root‐to‐shoot transport of K^+^. Using co‐transfection assays, gel electrophoresis mobility shift assays, and chromatin immunoprecipitation assays, we demonstrate that NLP7 directly interacts with and activates the promoters of these two genes. Furthermore, the enhanced K^+^ uptake and improved K deficiency responses driven by *NLP7* overexpression were mitigated in the *hak5* and *skor* mutants, demonstrating the significant physiological role of NLP7‐induced activation of these genes. Although *HAK5* and *SKOR* responded differently to K deficiency, both genes were activated similarly by NLP7 under K‐sufficient and K‐deficient conditions. Together, these findings suggest that K deficiency‐induced regulation and nitrate‐responsive regulation operate through independent transcriptional mechanisms but collaborate to ensure appropriate K acquisition under diverse N and K conditions, especially when K availability is limiting.

## Significance Statement

Balancing nitrogen (N) and potassium (K) acquisition is vital for plant growth, yet the mechanisms that maintain this balance are not well understood. We show that NIN‐LIKE PROTEIN7 (NLP7), which acts as a nitrate sensor and a master regulator responsible for nitrate‐induced gene expression, directly activates *HAK5* (for K^+^ uptake from soil) and *SKOR* (for root‐to‐shoot transport of K^+^). Through nitrate signaling‐dependent activation of these genes, NLP7 assists in K acquisition under K‐deficient conditions, thereby improving growth and resistance to K limitation.

## INTRODUCTION

Potassium (K) is an essential plant nutrient and a key fertilizer component, alongside nitrogen (N) and phosphorus (P). K^+^ is the most abundant cation in plants, constituting about 2–10% of their dry weight (Clarkson & Hanson, 1980; Wang et al., 2022), and supports numerous vital physiological processes. It activates a wide range of cytoplasmic enzymes (Leigh & Wyn Jones, 1984; Wang & Wu, 2010), regulates osmotic and turgor pressure, maintains membrane potential and pH homeostasis, and balances cytoplasmic anion charge (Clarkson & Hanson, 1980). Under sufficient K^+^ availability, cytoplasmic K^+^ levels are maintained at 100–200 mM (Clarkson & Hanson, 1980; Leigh & Wyn Jones, 1984; Walker et al., 1996). When K^+^ availability decreases, cytoplasmic K^+^ levels fall, triggering K deficiency symptoms and adaptive responses. These include leaf chlorosis (Feng et al., 2021; Xu et al., 2006), inhibited primary root elongation (Kellermeier et al., 2013), fewer lateral roots (Shin & Schachtman, 2004), enhanced root hair development (Desbrosses et al., 2003; Høgh‐Jensen & Pedersen, 2003), and increased root expression of high‐affinity K^+^ transporter genes (Bañuelos et al., 2002; Gierth et al., 2005; Wang et al., 1998). Since soil K^+^ is typically below 1 mM (Luan et al., 2009), plants often experience K deficiency in natural ecosystems. Consequently, K fertilization is essential in agricultural systems to ensure adequate K^+^ supply and to maintain crop productivity (Zörb et al., 2014).

Plants absorb K^+^ from the soil and distribute it internally *via* specific K^+^ transporters. In *Arabidopsis thaliana* (Arabidopsis), two principal transporters (the ARABIDOPSIS K^+^ TRANSPORTER 1 (AKT1) from the Shaker family and the HIGH‐AFFINITY K^+^ TRANSPORTER 5 (HAK5) from the KUP/HAK/KT family) play primary roles in root K^+^ uptake (Lara et al., 2020; Nieves‐Cordones et al., 2016; Rubio et al., 2008), although KUP7 of the KUP/HAK/KT family and the cation/proton exchanger CHX13 also support root K^+^ uptake (Han et al., 2016; Zhao et al., 2008). In the *akt1 hak5* double‐knockout mutant, high‐affinity K^+^ uptake is reduced to approximately 15% of wild‐type (WT) levels (Pyo et al., 2010). *AKT1* is constitutively expressed in root hairs, epidermal cells, and cortical and endodermal cells of mature roots and encodes an inwardly rectifying K^+^ channel (Lagarde et al., 1996). *HAK5*, by contrast, is primarily expressed in the epidermis of lateral and primary roots and encodes a high‐affinity K^+^ transporter (Gierth et al., 2005). However, HAK5, rather than AKT1, is critical for K^+^ uptake under lower external K^+^ concentrations (below 20 μM) (Pyo et al., 2010; Rubio et al., 2008). *AKT1* and *HAK5* expression are regulated differently in response to K nutritional status. *AKT1* expression remains unchanged under K deficiency (Lagarde et al., 1996), whereas *HAK5* expression is strongly induced when K becomes deficient, enhancing K^+^ uptake (Gierth et al., 2005). This *HAK5* induction is mediated by MYB77 and RAP2.11 (Feng et al., 2021; Kim et al., 2012), while ARF2 suppresses *HAK5* expression under K‐sufficient conditions (Zhao et al., 2016). Both HAK5 and AKT1 are also regulated post‐translationally through phosphorylation. Under low K conditions, calcium sensor proteins CALCINEURIN B‐LIKE 1 (CBL1) and CBL9 are activated (Li et al., 2023) and, in turn, phosphorylate CBL‐INTERACTING PROTEIN KINASE 23 (CIPK23). The resulting CBL1/9‐CIPK23 complexes phosphorylate HAK5 and AKT1 on the plasma membrane to activate them under K deficiency (Li et al., 2023; Ragel et al., 2015; Xu et al., 2006). In addition to CIPK23, CIPK1 and CIPK9 have been suggested to activate HAK5 through phosphorylation (Lara et al., 2020).

Loading K^+^ into the xylem is an essential step for root‐to‐shoot translocation of K^+^. In Arabidopsis, this step is primarily mediated by two proteins: NITRATE TRANSPORTER 1.5 (NRT1.5 or NPF6.3), a proton‐coupled cation antiporter for the root‐to‐shoot transport of K^+^ and nitrate (NO_3_^−^) (Lin et al., 2008) and STELAR K^+^ OUTWARD RECTIFIER (SKOR), a Shaker family K^+^ channel that facilitates K^+^ efflux from root inner sheath cells and parenchyma cells to the xylem (Gaymard et al., 1998). Consistent with this, K^+^ accumulation in the *skor* mutant shoot is reduced to approximately 50% of that in WT (Gaymard et al., 1998). Under low K conditions, expression of both *NRT1.5* and *SKOR* is reduced, reducing K^+^ transport to the shoot and maintaining high K^+^ concentrations in root cells to preserve membrane potential (Du et al., 2019; Pilot et al., 2003). Their distinct functional roles have been clarified further: NRT1.5 plays a crucial role when external NO_3_^−^ concentrations are low, whereas SKOR becomes essential when NO_3_^−^ is excessive relative to K^+^ availability (Drechsler et al., 2015).

N is another macronutrient required in large quantities and the balance between N and K acquisition strongly affects crop yield (Brennan & Bolland, 2009; Coskun et al., 2017). Plants primarily absorb N as nitrate or ammonium (NH_4_^+^), but these two chemical forms have different effects on K^+^ uptake. A strong positive correlation between NO_3_^−^ uptake and K^+^ uptake in plants has been recognized for decades (Blevins et al., 1974; Fang et al., 2020). For example, wheat and barley seedlings treated with 1 mM KNO_3_ showed a significant increase in K^+^ uptake (Blevins et al., 1974), and NO_3_^−^ application under N‐deficient conditions altered the expression of multiple K^+^ transporter genes in tomato (Wang et al., 2001). In contrast, NH_4_^+^, which is similar to K^+^ in terms of hydration diameter, charge, and effects on membrane potential (Wang et al., 1996), inhibits K^+^ uptake in tobacco, barley, and Arabidopsis (Hoopen et al., 2010; Scherer et al., 1984). NH_4_^+^ also suppresses the upregulation of *HAK5* expression under low K conditions in Arabidopsis (Rubio et al., 2008). Taken together, these findings suggest that although both NO_3_^−^ and NH_4_^+^ serve as major N sources, only NO_3_^−^ uptake synchronizes with K^+^ uptake to maintain electrical neutrality (Delaire et al., 2014; Fang et al., 2020). Supporting this, a recent phenome analysis of Arabidopsis ecotypes revealed a significant correlation between K^+^ and NO_3_^−^ concentrations but not between K^+^ and NH_4_^+^ concentrations (Sugimura et al., 2025). At the post‐translational level, CIPK23 phosphorylates NRT1.1, enhancing NO_3_^−^ uptake under low NO_3_^−^ conditions (Ho et al., 2009), and phosphorylates NH_4_^+^ transporters, reducing NH_4_^+^ uptake (Straub et al., 2017). Despite these insights, the mechanism underlying transcriptional regulation coordinating N and K acquisition remains unresolved.

In NO_3_^−^ signaling, NIN‐LIKE PROTEIN (NLP) transcription factors act as both NO_3_^−^ sensors and master regulators of NO_3_^−^‐induced gene expression (Chardin et al., 2014; Konishi & Yanagisawa, 2014; Liu et al., 2022). NLPs are activated *via* direct NO_3_^−^ binding and NO_3_^−^‐dependent phosphorylation (Liu et al., 2017, 2022), after which they activate not only genes involved in N uptake and N assimilation but also those involved in other physiological processes, including N deficiency and phosphate starvation responses (Kiba et al., 2018; Ueda et al., 2020), phytohormone responses (Abualia et al., 2022; Maeda et al., 2018), and light utilization (Ariga et al., 2022). In this study, we investigated whether NO_3_^−^ signaling also influences K acquisition and K deficiency responses, with the goal of uncovering novel mechanisms that balance N and K acquisition.

## RESULTS

### Reduced K^+^ uptake in the *nlp6/7* mutant

NLP7 is the predominant transcription factor mediating NO_3_^−^ signaling and regulating NO_3_^−^ uptake in Arabidopsis and NLP6 plays an auxiliary role (Castaings et al., 2009; Cheng et al., 2023; Guan et al., 2017; Konishi et al., 2021; Konishi & Yanagisawa, 2013). To investigate the effect of NLP6/7‐mediated NO_3_^−^ signaling on K^+^ uptake, we measured K^+^ uptake in the *nlp6nlp7* double mutant using ^86^Rb uptake as an indicator. Rb^+^ and K^+^ are thought to be absorbed and transported within plants *via* a common transport system. ^86^Rb is more commonly used instead of ^42^K to analyze K absorption dynamics in plants because ^42^K has a short half‐life (Kanno et al., 2023; Läuchli & Epstein, 1970; Osakabe et al., 2013; Zhao et al., 2008). ^86^Rb^+^ uptake was reduced in roots of *nlp6nlp7* seedlings adapted to low K conditions to about 20% of the activity in the WT seedlings (Figure 1), indicating that NLP6/7‐mediated NO_3_^−^ signaling promotes K^+^ uptake.

**Figure 1:**
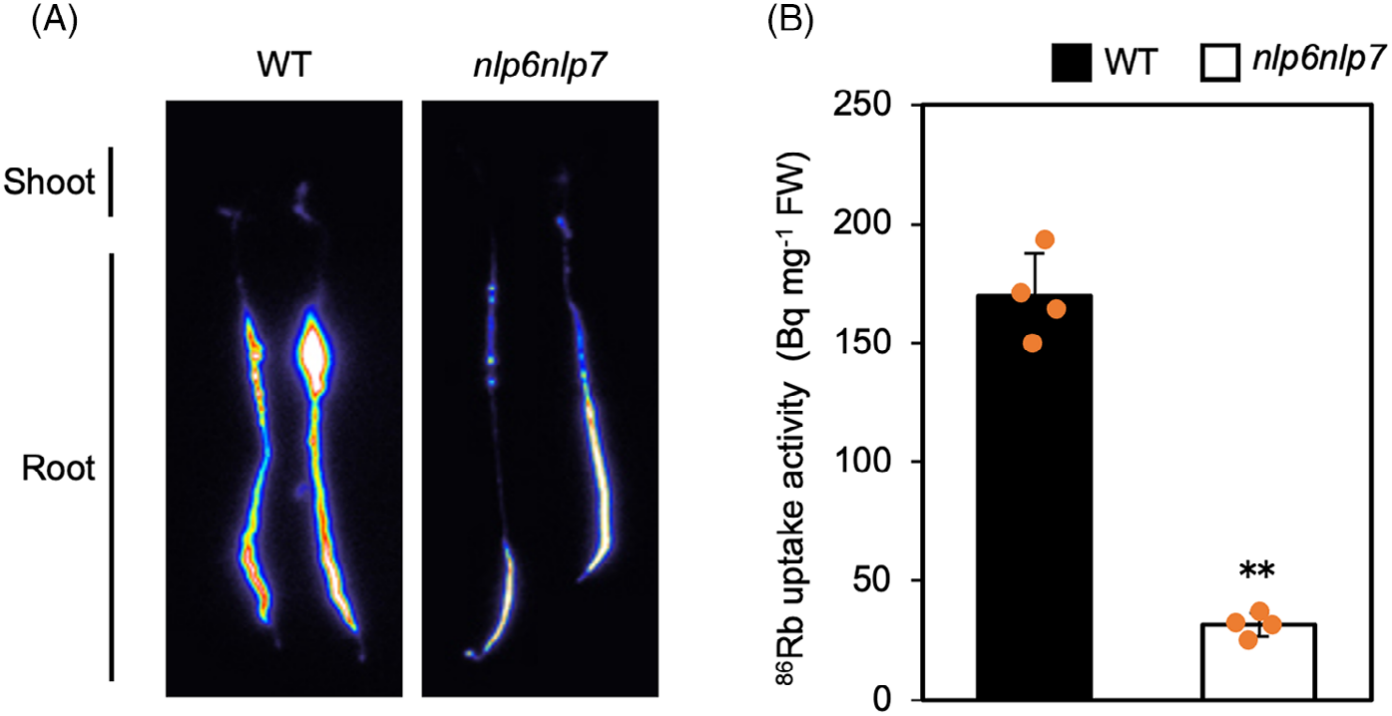
^86^Rb uptake in WT and *nlp6nlp7* seedlings. (A) Images of wild‐type (WT) and *nlp6nlp7* seedlings showing ^86^Rb uptake. The seedlings were exposed to low K conditions for 4 days and then transferred to a K‐free liquid nutrient solution containing ^86^RbCl to absorb ^86^Rb for 20 min. (B) Quantification of ^86^Rb absorbed. Data are shown as mean ± SD (*n* = 4 biological repeats). Statistical significance between WT and *nlp6nlp7* seedlings was determined using Student's *t*‐test (**P < 0.01). FW, fresh weight.

### Altered susceptibility to K deficiency in *nlp7, nlp6nlp7*, and *NLP7* ‐OX seedlings

Next, we explored the physiological significance of NLP6/7‐nitrate signaling in K deficiency response by comparing seedling growth among WT Arabidopsis, the *NLP7* knockout mutant (*nlp7*), the *nlp6nlp7* double mutant, and the *NLP7*‐overexpressing line (NLP7‐OX) under K‐sufficient and K‐deficient conditions. Seedlings were grown for 7 days under K‐sufficient (5 mM K^+^) or low K (2 μM K^+^) conditions after a 5‐day cultivation on half‐strength Murashige‐Skoog salts‐containing medium (1/2 MS medium). Growth was almost comparable among all seedlings under K‐sufficient conditions (Figure 2), although *nlp7* and *nlp6nlp7* seedlings showed slight reductions in shoot and root growth, possibly due to reduced N assimilation (Figure 2C–F). The *nlp7* and *nlp6nlp7* seedlings showed pronounced growth defects under low K conditions, with significant decreases in *F*v/*F*m ratio (the maximum efficiency of photosystem II), fresh shoot weight, primary root length, and fresh root weight, compared with WT seedlings, while the *NLP7*‐OX seedlings exhibited milder growth defects. These results revealed that loss of *NLP7* renders Arabidopsis seedlings hypersensitive to K deficiency whereas *NLP7* overexpression confers hyposensitivity. Furthermore, *nlp6nlp7* seedlings showed more severe growth defects than *nlp7* seedlings, suggesting the redundant role of NLP6 and NLP7 in determining susceptibility to K deficiency. We obtained similar results with hydroponically cultivated seedlings, along with an additional finding: significant leaf necrosis in *nlp6nlp7* seedlings grown under K‐deficient conditions (Figure 3).

**Figure 2:**
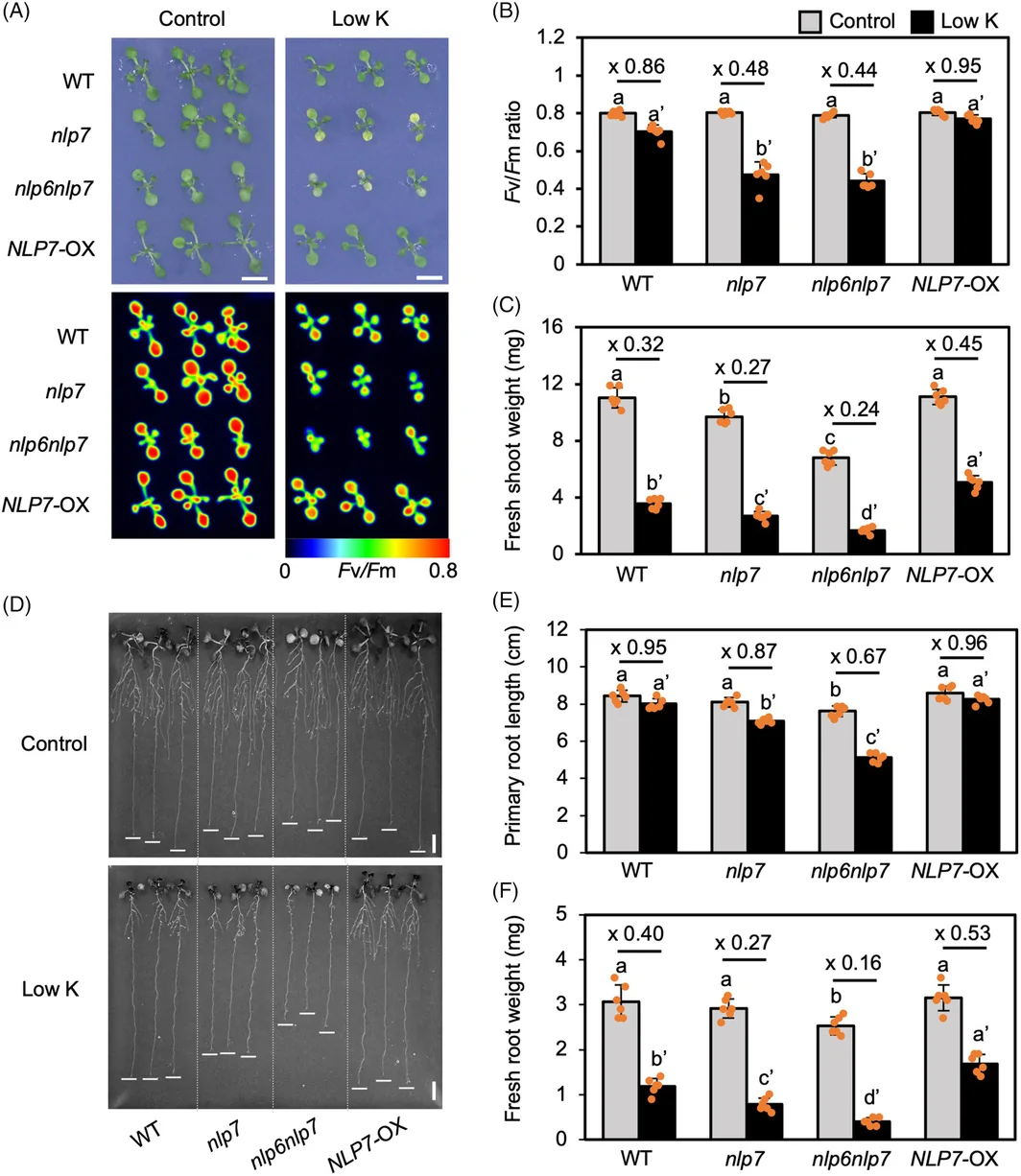
Altered susceptibility to K deficiency in *nlp7*, *nlp6nlp7*, and *NLP7*‐overexpressing seedlings. (A) and (D) show images of shoot and *F*v/*F*m (A) and root (D) of WT, *nlp7*, *nlp6nlp7*, and *NLP7*‐overexpressing (*NLP7*‐OX) seedlings. *F*v/*F*m ratio is represented in (B). Fresh shoot weight (C), primary root length (E), and fresh root weight (F) were measured, and the data are shown as mean ± SD (*n* = 8 biological repeats). Positions of root tips are indicated with white bars in (D). The seedlings were grown on 1/2 MS medium for 5 days and then exposed to K‐sufficient or low K conditions for 7 days. Different letters indicate significant differences between mean values as determined by Tukey's multiple comparison test at *P* < 0.05. Scale bar, 10 mm (A, D).

**Figure 3:**
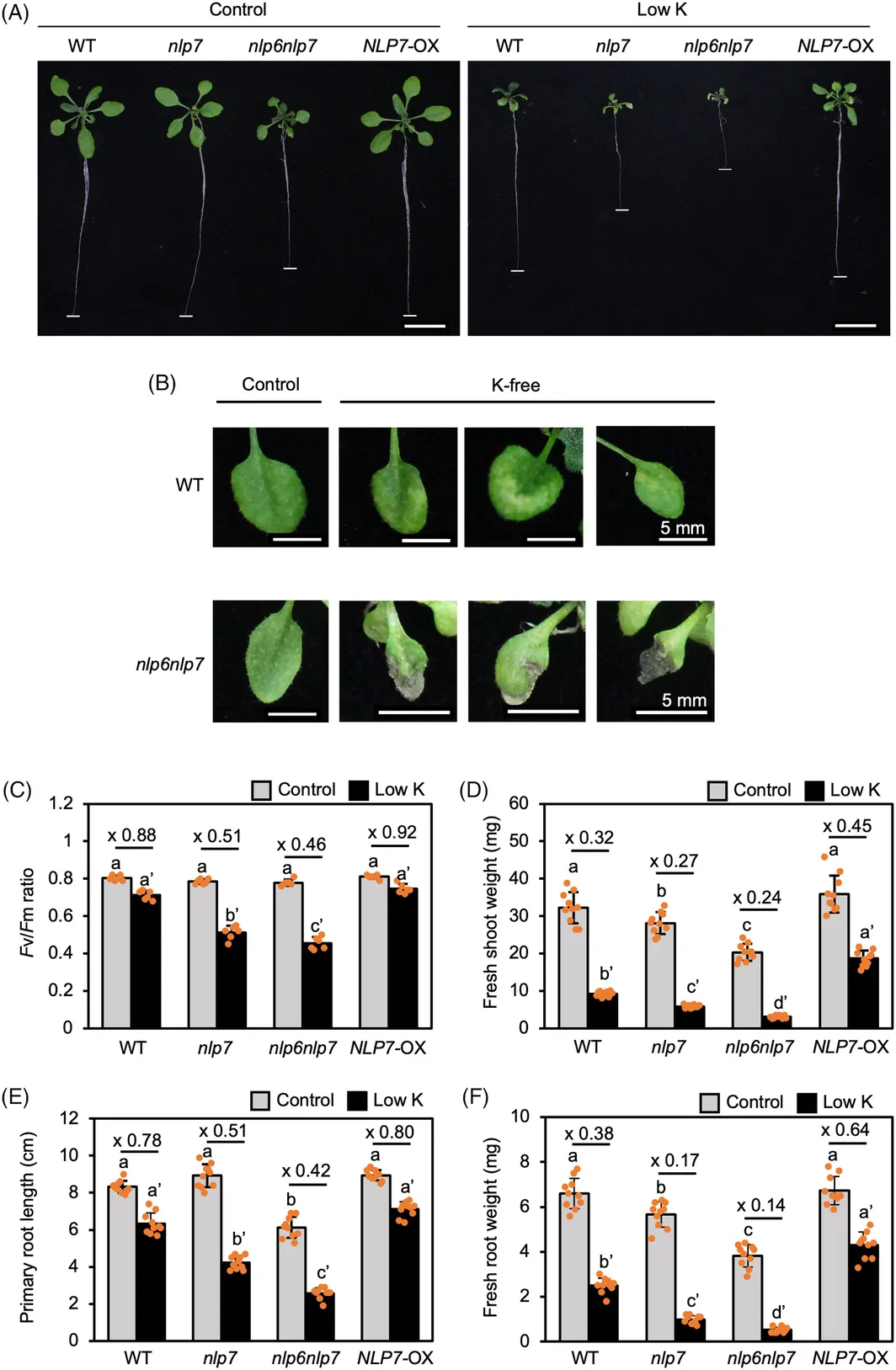
Susceptibility to K deficiency in WT, *nlp7*, *nlp6nlp7*, and the *NLP7*‐OX seedlings under hydroponic growth conditions. Full body (A) and enlarged leaf (B) images of WT, *nlp7*, *nlp6nlp7*, and the *NLP7*‐OX seedlings. Positions of root tips are indicated with white bars in (A). *F*v/*F*m ratios are shown in (C). Seedlings were grown in K‐sufficient nutrient solution for 12 days and then transferred to K‐sufficient (1 mM KCl) or K‐free nutrient solution for 7 days. Fresh shoot weight (D), primary root length (E), and fresh root weight (F) were measured, and the data are shown as mean ± SD (*n* = 6 biological repeats). Different letters indicate significant differences between mean values as determined by Tukey's multiple comparison test at *P* < 0.05 (one‐way ANOVA). Scale bar, 30 mm (A) and 5 mm (B).

### Effects of impaired NO_3_ ^−^ signaling on K deficiency‐induced gene expression

To elucidate why loss of NLP6/7‐mediated NO_3_^−^ signaling rendered Arabidopsis seedlings hypersensitive to K deficiency, we examined changes in root gene expression between WT and *nlp6nlp7* seedlings before and after exposure to K deficiency. Comparisons of data obtained before K deprivation (0DT roots) and after 4 days of deprivation (4DT roots) revealed that K deficiency led to the upregulation of 98 genes (fold change [FC] > 2, *P*‐value <0.05) and the downregulation of 59 genes (FC < 0.5, *P*‐value <0.05) in WT roots (Figure 4A). Among these genes, 22 genes were upregulated by more than fourfold, while only two genes were downregulated to less than one fourth (Tables S1 and S2). The genes that were highly upregulated included *HAK5*, *VSP1/2* (jasmonic acid‐related vegetative storage protein genes), and *NITRATE TRANSPORTER 2.1* (*NRT2.1*), which encodes the principal high‐affinity NO_3_^−^ transporter (Table S1). This observation was consistent with previous results (Armengaud et al., 2004). Next, we investigated the effects of impaired NO_3_^−^ signaling by comparing gene expression levels in WT and *nlp6nlp7* roots. In *nlp6nlp7* roots, only five genes were upregulated before K deprivation and three after K deprivation compared with WT roots (Figure 4A; Tables S3 and S5). In contrast, 62 genes were downregulated before K deprivation and 41 after K deprivation (Figure 4A; Tables S4 and S6). Among the 98 genes upregulated by K deficiency treatment, the expression of 26 genes was altered (either upregulated or downregulated) in *nlp6nlp7* roots compared with WT roots. Similarly, the expression of 15 of the 59 genes downregulated by K deficiency was modified in *nlp6nlp7* roots (Figure 4A). These results suggest that NLP6/7‐mediated NO_3_^−^ signaling may influence some, but not all, aspects of K deficiency‐responsive gene expression.

**Figure 4:**
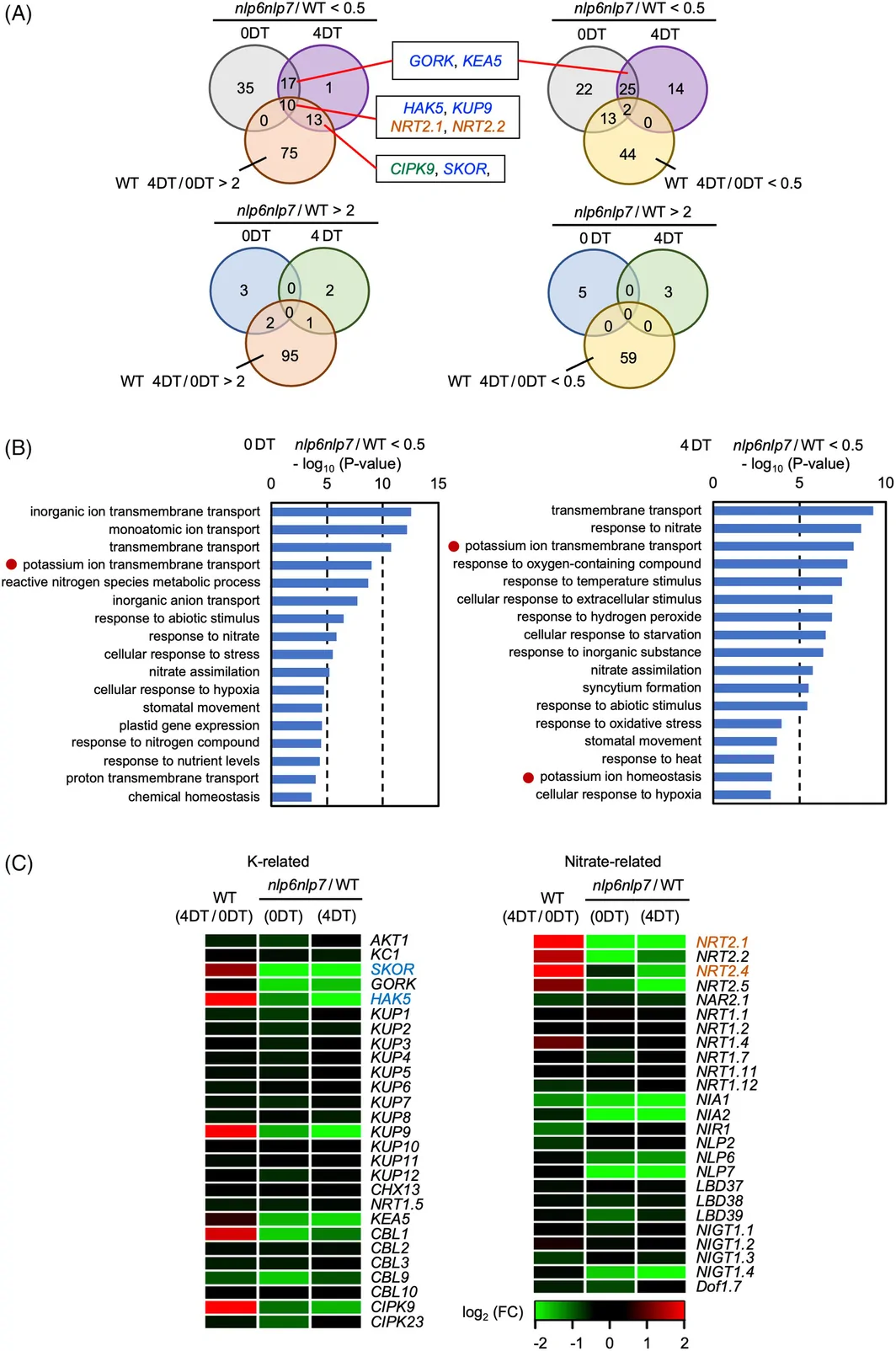
Relationship between K deficiency‐responsive genes and genes regulated by NLP6/7‐mediated signaling. (A) Venn diagrams showing the number of genes with altered expression (decreased: *nlp6nlp7*/WT <0.5; increased: *nlp6nlp7*/WT >2) due to the disruption of *NLP6* and *NLP7* at the start (0DT) and after 4 days of K deficiency treatment (4DT), as well as genes with altered expression after 4 days of K deficiency (decreased: 4DT/0DT <0.5; increased: 4DT/0DT >2). Genes associated with K transporter, K deficiency regulation, and nitrate transport are indicated in blue, green, and red, respectively. (B) GO biological terms overrepresented among genes with reduced expression (<0.5‐fold decrease) in *nlp6nlp7* seedlings vs. WT at 0DT and 4DT. Red circles highlight two K‐related GO terms. (C) Heatmaps indicating how K deficiency and the disruption of *NLP6* and *NLP7* affect the expression of K‐related and nitrate‐related genes. For each gene, log_2_ expression ratios are shown for WT (4DT/0DT) and for *nlp6nlp7* compared with WT (*nlp6nlp7*/WT) at 0DT and 4DT, as indicated by the color scale. FC: fold change, DT: days of treatment.

### Independent control of common target genes by K deficiency‐responsive regulation and NLP6/7‐mediated regulation

To clarify the effects of NLP6/7‐mediated NO_3_^−^ signaling on the expression of K‐related genes, we performed gene ontology (GO) analysis. Consistent with the established roles of NLP6 and NLP7 as transcriptional activators (Yanagisawa, 2026), a substantial number of genes were downregulated in *nlp6nlp7* roots. By contrast, only five genes under K‐sufficient and three genes under low K conditions were upregulated in *nlp6nlp7* roots (Figure 4A). Therefore, GO analysis focused on the genes downregulated in *nlp6nlp7* roots. This analysis revealed that NLP6/7‐mediated NO_3_^−^ signaling preferentially activates genes in K^+^ transmembrane transport and K^+^ homeostasis, in addition to genes in N‐related categories (Figure 4B).

Next, we investigated the effects of NLP6/7‐mediated NO_3_^−^ signaling on expression levels of K^+^ transport and K^+^ homeostasis‐related genes using heat map analysis. Both K deficiency‐inducible genes (such as *HAK5*, *KUP9*, and *CIPK9*) and K deficiency‐non‐inducible genes (such as *SKOR*, *KEA5*, and *GORK*) were downregulated in *nlp6nlp7* roots, largely independent of K status (Figure 4C, left panel). This pattern indicates that K deficiency‐responsive regulation and NLP6/7‐mediated transcriptional regulation act independently of their shared target genes. A similar separation of regulatory pathways was observed in N‐related gene expression. K deficiency increased expression levels of several high‐affinity NO_3_^−^ transporter genes (*NRT2.1*, *NRT2.2*, and *NRT2.4*) but did not activate other NLP target genes associated with NO_3_^−^ signaling and assimilation, such as NO_3_^−^ reductase genes (*NIA1/2*) (Figure 4C, right panel). Notably, decreases in expression levels of several known NLP7 target genes, such as *NIR*, were not detected in *nlp6nlp7* roots in this experiment, probably because of the constant supply of N sources.

### NLP6/7‐dependent and NO_3_ ^−^‐responsive activation of *HAK5* , *SKOR* , and *NRT2.1* expression

Genes that are strongly downregulated in *nlp6/7* roots represent strong candidates for direct NLP6/7 target genes. Among these were *HAK5*, a key gene for K^+^ uptake under low K^+^ conditions (Pyo et al., 2010), and *SKOR*, a critical gene for root‐to‐shoot transport of K^+^ (Gaymard et al., 1998) (Figure 4C). We therefore quantified the expression levels of *HAK5* and *SKOR* in WT, *nlp6nlp7* and *NLP7*‐OX roots using reverse transcription‐quantitative PCR (RT‐qPCR). We also examined *NRT2.1*, another candidate gene for regulation by both NLP6/7 and K deficiency based on our results. All three genes showed reduced expression in *nlp6nlp7* roots but were elevated in *NLP7*‐OX roots regardless of K deficiency (Figure 5A). Although *NLP7* overexpression increased the expression levels of all three genes, K deficiency produced gene‐specific effects: it increased *HAK5* and *NRT2.1* expression but reduced *SKOR* expression. This reduction in *SKOR* expression under low K conditions was more pronounced in RT‐qPCR analysis than in transcriptome analysis and is consistent with previous results (Du et al., 2019; Pilot et al., 2003). Together, these results suggest *HAK5* and *SKOR* as previously unidentified NLP6 and NLP7 target genes. They demonstrate that K deficiency and NO_3_^−^ signaling act through independent transcriptional mechanisms that share common target genes, generating distinct expression patterns in response to K and N nutrient status.

**Figure 5:**
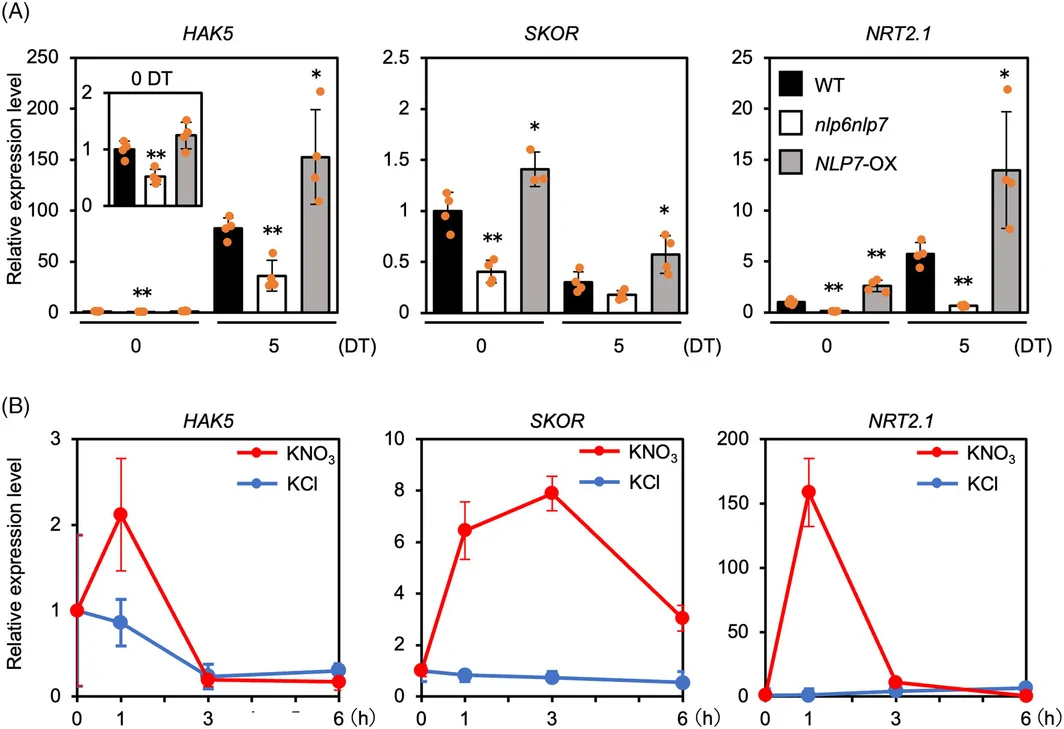
Changes in *HAK5*, *SKOR*, and *NRT2.1* expression in response to K deficiency and nitrate supply. (A) *HAK5*, *SKOR*, and *NRT2.1* expression levels in WT, *nlp6nlp7*, and *NLP7*‐OX seedlings before (0DT) and after 4 days of K deficiency treatment. (B) Time course analysis of nitrate‐induced expression of *HAK5*, *SKOR*, and *NRT2.1*. WT seedlings were grown for 5 days on modified 1/10 MS liquid medium containing only ammonium succinate as the N source and then treated with 10 mM KNO_3_ or 10 mM KCl (control) for the indicated periods. Expression levels were normalized to *UBQ10*, and the data are shown as mean ± SD (*n* = 4 biological repeats). In (A), values for WT expression before K deficiency treatment were set to 1. Asterisks above each bar indicate significant differences between WT and other samples (*P < 0.05, **P < 0.01; two‐tailed Student's *t*‐test).

We further examined the expression of *NRT2.1*, *HAK5*, and *SKOR* over time after supplying NO_3_^−^. All three genes showed similar temporal expression patterns with rapid initial increases, followed by a decline over time (Figure 5B). However, the peak of *SKOR* expression was delayed compared with *HAK5* and *NRT2.1* (Figure 5B). This is likely because nitrate concentrations rise more rapidly in root epidermal cells expressing *NRT2.1* and *HAK5* than in the root xylem parenchyma expressing *SKOR* (Gaymard et al., 1998; Gierth et al., 2005; Wirth et al., 2007).

### NLP7 directly activates the *HAK5* and *SKOR* promoters

We next investigated whether NLP7 directly activates *HAK5* and *SKOR*. We focused on NLP7 rather than NLP6 because NLP7 appears to play a more important role in regulating K deficiency responses (Figure 2), consistent with its role in other NO_3_^−^‐responsive processes. To test promoter activation, we performed co‐transfection assays in Arabidopsis protoplasts employing an effector plasmid for *NLP7* expression and reporter plasmids in which the firefly luciferase gene (*LUC*) was driven by either the *HAK5* promoter (−1611 to +77 relative to the transcription start site) or the *SKOR* promoter (−1602 to +85) (Figure 6A). Reporter plasmids with *LUC* driven by the *35S* or *NRT2.1* promoter served as negative and positive controls, respectively. The results showed that NLP7 activated the *HAK5* and *SKOR* promoters *in vivo*. To understand the binding properties of these promoters *in planta*, we conducted a chromatin immunoprecipitation (ChIP) assay using *NLP7*‐OX seedlings. NLP7 showed strong binding to region **e** of the *HAK5* promoter and region **b** of the *SKOR* promoter (Figure 6B,C). Both regions contain DNA sequences resembling the semi‐palindromic NLP‐binding consensus sequence, TG(A/G)C(C/T)CTT‐N_10_‐AA(G/A)(A/G)GGCA, either half of which may be sufficient for NLP‐binding (Konishi & Yanagisawa, 2014; Yanagisawa, 2026; Figure 6B,C).

**Figure 6:**
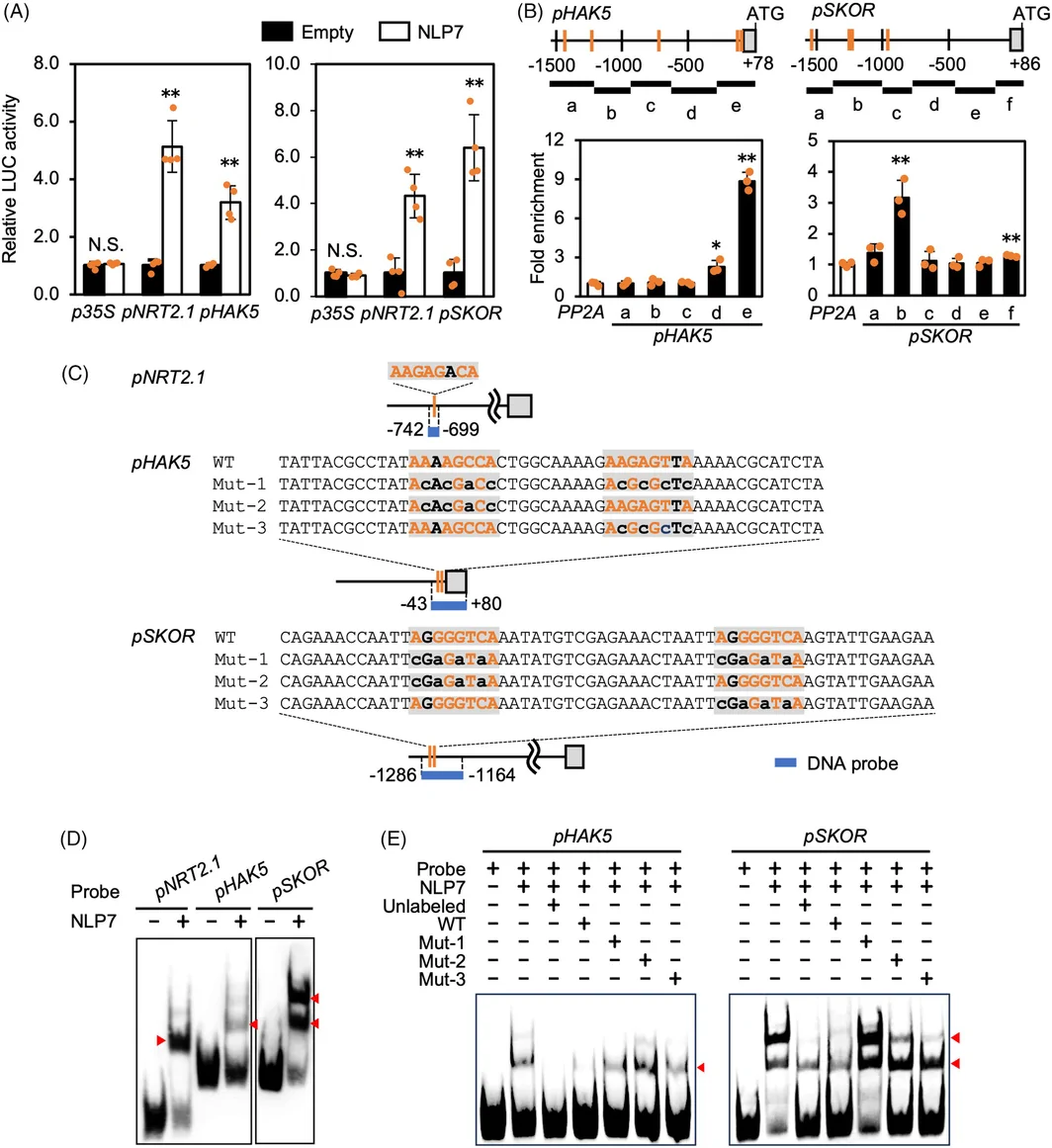
Binding and activation of the *HAK5* and *SKOR* gene promoters by NLP7. (A) Activation of *HAK5* and *SKOR* gene promoters by NLP7 *in vivo*. Reporter plasmids containing the *LUC* reporter gene driven by the *35S*, *NRT2.1*, *HAK5*, or *SKOR* promoter were co‐transfected into Arabidopsis protoplasts together with an NLP7 expression vector or empty vector (negative control) and an internal control plasmid (UBQ10‐GUS). LUC activity in each sample was normalized to GUS activity and expressed as a relative value to that from protoplasts co‐transfected with the p35S‐LUC reporter plasmid and the empty vector. (B) ChIP assays to analyze the *in vivo* binding of NLP7 to *HAK5* and *SKOR* promoters in *NLP7*‐OX seedlings. PCR‐amplified regions of immunoprecipitated DNA are shown as horizontal black bars in the *HAK5* and *SKOR* gene promoter structures with gray boxes indicating 5′‐untranslated regions and orange bars indicating putative NLP7‐binding motifs. Fold Enrichment of each promoter region was expressed relative to the negative control gene *PP2A*. (C) Positions of DNA probes (horizontal blue bars) and synthetic competitor DNAs used in EMSA. Gray boxes indicate 5′‐untranslated regions, and nucleotide numbers are relative to the transcription start site. The synthetic DNAs correspond to native regions of two putative NLP7‐binding motifs (WT) in the *HAK5* and *SKOR* promoters or mutated versions of these regions (Mut‐1, Mut‐2, and Mut‐3). Putative NLP7‐binding motifs are highlighted in gray, and nucleotides in the motifs that match and do not match the consensus sequence are indicated in orange and black, respectively. Mutated nucleotides are indicated in lowercase. (D) EMSA. Biotin‐labeled DNA probes were incubated in the presence (+) or absence (−) of NLP7 protein. Red arrows indicate protein–DNA complexes. (E) Competition assays. Unlabeled DNA probes or synthetic DNAs were added to binding reaction mixtures as competitors at a 50‐fold molar excess compared with the biotin‐labeled DNA probe.

We further assessed the binding using gel electrophoresis mobility shift assay (EMSA). Recombinant NLP7 protein directly bound to a DNA fragment (−43 to +80) from region **e** of the *HAK5* promoter and a DNA fragment (−1286 to −1164) from region **b** of the *SKOR* promoter, as well as to a control DNA fragment from the *NRT2.1* promoter containing an NLP‐binding site (Maeda et al., 2018) (Figure 6D). When the *SKOR* promoter fragment was used as a probe, two shifted bands were clearly detected. To confirm the sequence‐specific binding of NLP7 to the DNA probes, competition assays were carried out using unlabeled probes and 50‐bp or 60‐bp synthetic DNAs with native or mutant sequences (Figure 6C). Since the putative binding sites in the *HAK5* and *SKOR* promoters consisted of a tandem repeat of the DNA sequence motif resembling half of the semi‐palindromic NLP‐binding consensus sequence, mutations were introduced into either both motifs (Mut‐1) or one of the two motifs (Mut‐2 and Mut‐3). Unlabeled probes and synthetic DNAs with two native motifs (WT) competed with the labeled DNA probes, while synthetic DNAs with mutations in both motifs (Mut‐1) did not. Interestingly, synthetic DNAs with mutations in one of the two motifs (Mut‐2 and Mut‐3) did not compete with the labeled DNA probes in the NLP7‐*HAK5* probe interaction but partially competed in the NLP7–*SKOR* probe interaction (Figure 6E). Therefore, the two motifs in the *SKOR* promoter may independently interact with NLP7, whereas the two motifs in the *HAK5* promoter may be necessary for the interaction with NLP7. These results resemble the previously observed phenomenon: half of the semi‐palindromic NLP‐binding sequence solely acts as a nitrate‐responsive element (NRE) in some cases, while both halves of the semi‐palindromic NLP‐binding sequence are required to function as an NRE in other cases (Konishi & Yanagisawa, 2014; Yanagisawa, 2026).

### Contribution of NLP7‐mediated activation of *HAK5* and *SKOR* to enhancing K^+^ uptake and modulating K deficiency responses

To examine the physiological significance of NO_3_^−^‐dependent activation of *HAK5* and *SKOR*, we generated *NLP7‐*OX*/hak5* and *NLP7‐*OX*/skor* lines by cross‐breeding *NLP7*‐OX plants with the respective *hak5* and *skor* mutants. Consistent with the upregulation of *HAK5* and *SKOR* in the *NLP7‐*OX line (Figure 5B), *NLP7* overexpression promoted K^+^ uptake in WT seedlings (Figure 7). In contrast, K^+^ uptake was only minimally increased in *NLP7‐*OX*/hak5* seedlings, indicating that enhanced K^+^ uptake activity in the WT seedlings is largely attributable to the activation of *HAK5*. Although *NLP7* overexpression also increased K^+^ uptake activity in the *skor* mutant, K^+^ uptake in the *skor* mutant overexpressing *NLP7* (*NLP7*‐OX/*skor*) remained substantially lower than in the *NLP7‐*OX line (Figure 7). These results demonstrate that the increase in both *HAK5* and *SKOR* expression by NLP7‐mediated NO_3_^−^ signaling contributes to enhanced K^+^ uptake, but the activation of *HAK5* plays the predominant role in elevating K^+^ uptake activity.

**Figure 7:**
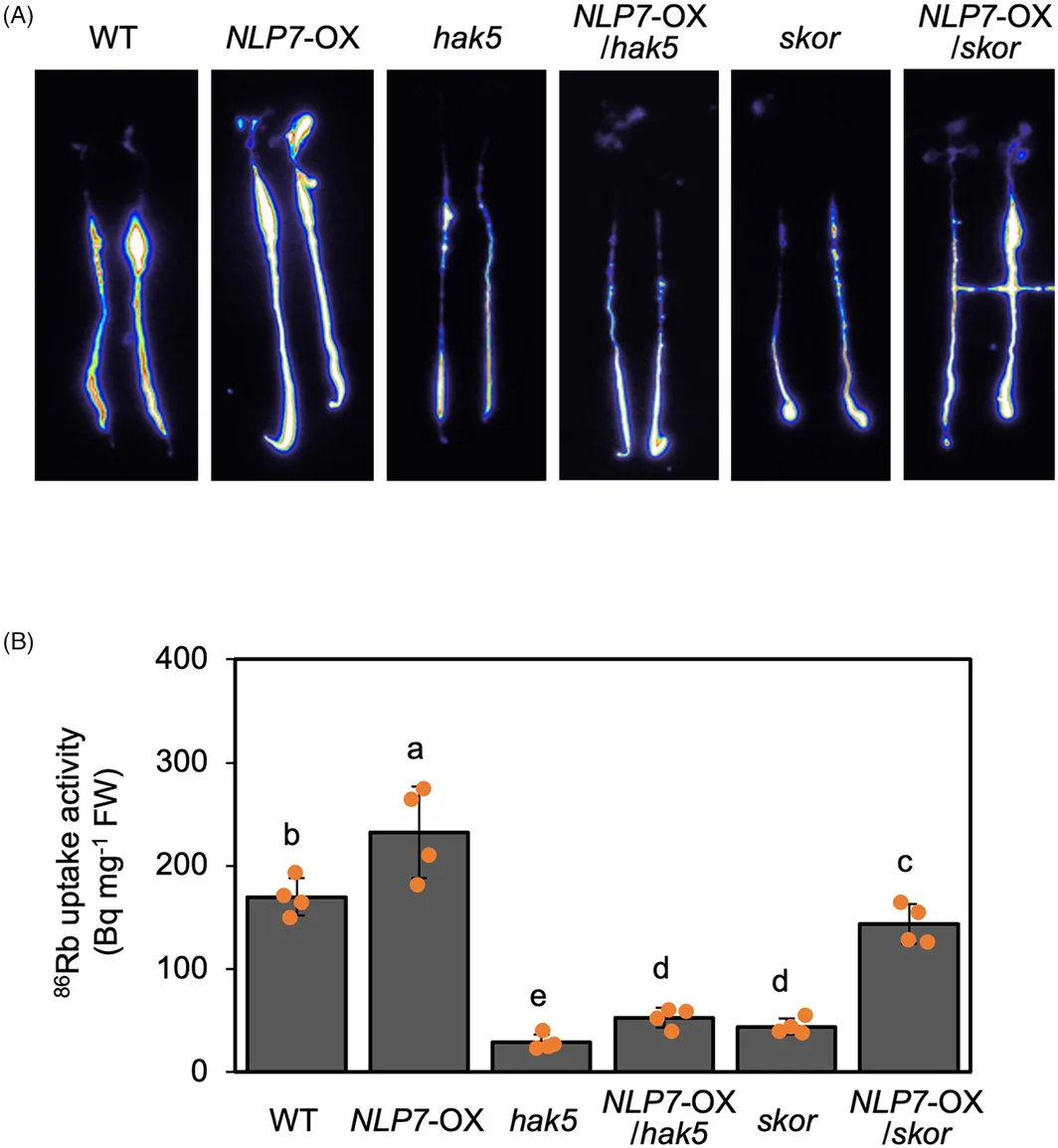
^86^Rb uptake in *NLP7*‐OX, *hak5*, *NLP7*‐OX/*hak5*, *skor*, and *NLP7*‐OX/*skor* seedlings. Images of ^86^Rb uptake (A) and quantification of ^86^Rb absorbed (B) in WT, *NLP7*‐OX, *hak5*, *NLP7*‐OX/*hak5*, *skor*, and *NLP7*‐OX/*skor* seedlings are shown. Six‐day‐old seedlings were exposed to low K conditions for 4 days and then transferred to a K‐free liquid nutrient solution containing ^86^RbCl. The data are shown as mean ± SD (*n* = 4 biological repeats). Different letters indicate significant differences between means as determined by Tukey's multiple comparison test at *P* < 0.05.

We next evaluated susceptibility to K deficiency in *NLP7‐*OX, *NLP7‐*OX*/hak5*, and *NLP7‐*OX*/skor* seedlings (Figure 8). Under K‐sufficient conditions, WT, *NLP7‐*OX, *hak5*, *NLP7‐*OX*/hak5*, *skor*, and *NLP7‐*OX*/skor* seedlings displayed comparable growth (Figure 8A, left panel). Under low K conditions, however, *NLP7‐*OX seedlings exhibited improved growth, whereas *hak5* and *skor* seedlings showed reduced growth compared with the WT seedlings. Notably, *NLP7* overexpression alleviated and partially restored growth defects in *hak5* and *skor* backgrounds (see *NLP7‐*OX*/hak5* and *NLP7‐*OX*/skor* seedlings) (Figure 8A, right panel). This effect was also reflected in improved *F*v/*F*m ratios, fresh shoot weights, primary root lengths, and fresh root weights in *hak5* and *skor* seedlings (Figure 8B–E). These results, together with the K^+^ uptake activity data, demonstrate that both NLP7‐mediated activation of *HAK5* and *SKOR* enhances tolerance to low K environments.

**Figure 8:**
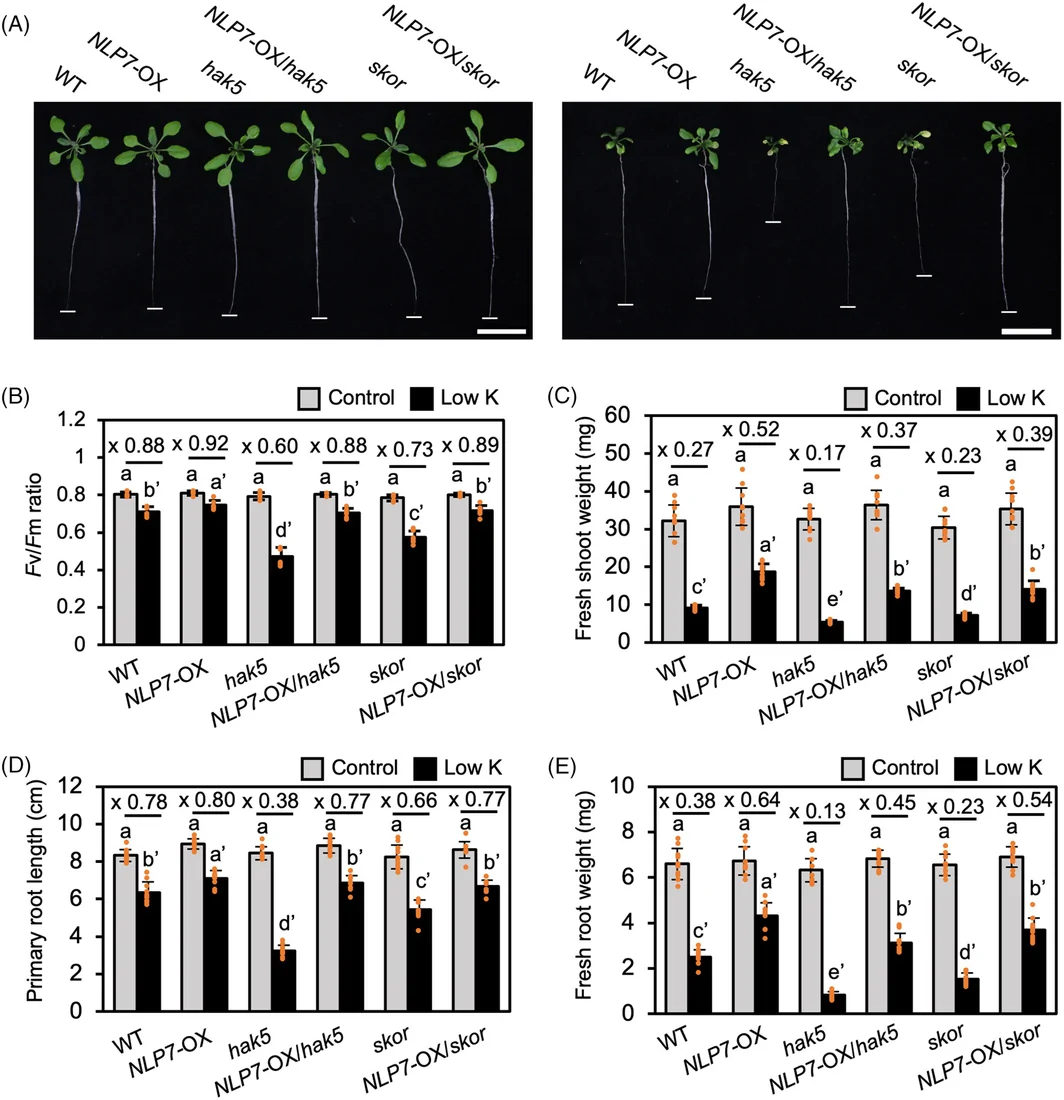
Susceptibility to K deficiency in *NLP7*‐OX, *hak5*, *NLP7*‐OX/*hak5*, *skor*, and *NLP7*‐OX/*skor* seedlings. Full body images of seedlings under K‐sufficient (left) and low K condition (right) (A), *F*v/*F*m ratios (B), fresh shoot weight (C), primary root length (D), and fresh root weight (E) of WT, *NLP7*‐OX, *hak5*, *NLP7*‐OX/*hak5*, *skor*, and *NLP7*‐OX/*skor* seedlings. Seedlings were hydroponically grown in K‐sufficient nutrient solution for 12 days and then transferred to either K‐sufficient (1 mM KCl) or K‐free nutrient solution for 7 days. Positions of root tips are indicated with white bars in (A). In (B–E), the data are shown as mean ± SD. (*n* = 6 biological repeats). Different letters indicate significant differences between mean values, as determined by Tukey's multiple comparison test at *P* < 0.05. Scale bar in (A), 40 mm.

## DISCUSSION

This study demonstrated that NLP6/7‐mediated NO_3_^−^ signaling activates *HAK5* and *SKOR*. Although transcriptional regulatory mechanisms underlying NLP6/7‐mediated activation and K deficiency response independently influence *HAK5* and *SKOR* expression, NLP6/7‐mediated activation is required for the full induction of these genes (Figure 5A), for high K^+^ uptake activity (Figures 1 and 7), and for improved growth (Figures 2, 3 and 8) under K‐deficient conditions. These findings reveal a novel mechanism that balances N and K acquisition, offering new insights into the molecular basis of balanced nutrient acquisition across diverse nutrient conditions.

### Physiological significance of NLP6/7‐mediated activation of *HAK5* , *SKOR* , and other K‐related genes

K^+^ regulates intracellular charge as the counterion to NO_3_^−^ and activates enzymes involved in NO_3_^−^ assimilation (Blevins et al., 1978; Coskun et al., 2017). Therefore, it is reasonable to assume that NLP6/7‐mediated NO_3_^−^ signaling synchronously upregulates both NO_3_^−^ uptake and K^+^ uptake to optimize growth. However, enhancing K^+^ uptake by NLP6/7‐mediated NO_3_^−^ signaling is more significant under low K conditions (Figures 2, 3 and 8), probably because sufficient K^+^ can be absorbed without increased K^+^ uptake activity when the environmental K^+^ concentration is high. This interpretation aligns with the fact that NLP6/7 target genes included *HAK5* but not *AKT1* (Figure 4). Although both AKT1 and HAK5 are responsible for K^+^ uptake from the soil, AKT1 can mediate K^+^ efflux when extracellular K^+^ concentration becomes lower than intracellular levels (Geiger et al., 2009). AKT1 alters its properties by forming a heterotetramer with KC1 (a Shaker family protein) to prevent the backflow of K^+^ into the extracellular space under low K conditions (Geiger et al., 2009). Hence, selective upregulation of *HAK5* expression, but not *AKT1*, by NLP6/7‐mediated nitrate signaling is consistent with the physiological requirements.

NLP7 directly activates the *SKOR* promoter as well as the *HAK5* promoter. SKOR is responsible for loading K^+^ into the xylem, where K^+^ and NO_3_^−^ act as the primary counterion pair, as in the cytoplasm (Drechsler et al., 2015; Engels & Marschner, 1993). This vital role of SKOR is underscored by the approximately 50% reduction in shoot K^+^ accumulation in the *skor* mutant (Gaymard et al., 1998). Thus, NLP6/7‐mediated activation of *SKOR* likely plays a crucial role in both balancing NO_3_^−^ and K^+^ concentrations in the xylem and promoting root‐to‐shoot transport of these ions. This hypothesis is supported by the reduced effect of *NLP7* overexpression on K^+^ uptake and K deficiency responses in the *skor* background (Figures 7 and 8). While NLP6/7‐mediated NO_3_^−^ signaling promotes K^+^ uptake from the soil through *HAK5*, SKOR‐mediated translocation of K^+^ may function as a pump that lowers K^+^ concentration in the root.

Intriguingly, *SKOR* expression was downregulated under low K conditions, likely because less K^+^ needs to be transported to the shoot when K^+^ uptake is limited. However, *NLP7* overexpression enhances *SKOR* expression even under low K conditions, suggesting that *SKOR* upregulation must increase in proportion to the increases in K^+^ uptake induced by NLP6/7‐mediated activation of *HAK5*. Thus, NLP6/7‐mediated activation of *HAK5* and *SKOR* may be the mechanism for fine‐tuning the amount of K^+^ translocated to the shoot and a rational strategy to support physiological processes in the shoot. The ability to independently regulate K^+^ uptake from the soil and its translocation may be essential for plants to adapt optimally to diverse K‐deficient environments.

Although changes in *HAK5* and *SKOR* expression in the *nlp7*, *nlp6nlp7*, and *NLP7*‐OX seedlings clearly affected K^+^ uptake activity and susceptibility to K deficiency, additional K deficiency‐inducible genes, such as *KUP9*, *KEA5*, and *CIPK9*, were also found as putative target genes of NLP6/7‐mediated NO_3_^−^ signaling (Figure 4). Further analysis of these genes may reveal additional mechanisms underscoring the interplay between K and N acquisition and between K deficiency and N responses (Figure 4C). KUP9 is an endoplasmic reticulum (ER)‐localized protein that directly mediates K^+^ and auxin efflux from the ER to the cytosol to maintain K^+^ and auxin homeostasis in the quiescent center cells under low K conditions (Zhang et al., 2020), and KEA5 is involved in pH and K^+^ homeostasis in endomembrane compartments (Zhu et al., 2018). Thus, NO_3_^−^ signaling may affect the intracellular distribution of K^+^, modulating K deficiency responses. *CIPK9*, which encodes a protein kinase that regulates the HAK5 activity *via* phosphorylation (Lara et al., 2020), may amplify the effects caused by NO_3_^−^ signaling on K^+^ uptake and the response to K deficiency. Further analysis of these genes may reveal additional mechanisms underlying these responses.

### Two independent mechanisms collaborate to coordinate the synchronous expression of K deficiency‐responsive genes and NO_3_ ^−^‐responsive genes

Only a fraction (about 25%) of K deficiency‐responsive genes were downregulated in the *nlp6nlp7* mutant, indicating that NLP6/7 regulates only some aspects of K deficiency responses. Conversely, only a fraction of NLP6/7‐regulated genes were K deficiency‐responsive, highlighting a partial overlap between nitrate‐ and K deficiency‐responsive genes (Figure 4A). These findings indicate the existence of two independent regulatory mechanisms governing K deficiency‐ and NO_3_^−^‐responsive gene expression with a group of shared target genes. As shown in this study, NLP6/7‐mediated NO_3_^−^ signaling enhanced *HAK5* and *SKOR* expression, whereas K deficiency differently affected *HAK5* and *SKOR* expression (Figure 5B). Similarly, K deficiency specifically enhanced the expression of high‐affinity NO_3_^−^ transporter genes such as *NRT2.1* and *NRT2.4*, but did not affect the expression of other N‐related genes (Figure 5B) and even slightly decreased the expression of genes involved in NO_3_^−^ reduction and assimilation, including the NO_3_^−^ reductase gene (*NIA1*) and the nitrite reductase gene (*NIR*). Taken together, these findings suggest that the collaborative action of K deficiency‐related regulation and NO_3_^−^‐induced regulatory mechanisms is responsible for maintaining ionic homeostasis between K^+^ and NO_3_^−^ under K‐deficient conditions. By enabling NO_3_^−^‐ uptake in proportion to increased K^+^ uptake activity, this coordination helps maintain the ionic balance. The primary reason why K^+^ uptake and NO_3_^−^ uptake are regulated in a coordinated manner is likely to maintain the ionic balance.

The mechanism by which the expression levels of *NRT2.1* and *NRT2.4* are increased in response to K deficiency remains unknown. MYB77 and RAP2.11 have been previously shown to regulate the K deficiency‐inducible expression of *HAK5* (Feng et al., 2021; Kim et al., 2012). It is important to investigate whether these transcription factors also regulate *NRT2.1* and *NRT2.4*. Elucidating the mechanism underlying the downregulation of *NRT2.1* and *NRT2.4* in response to K deficiency will shed light on the sophisticated regulatory mechanisms that balance nutrient acquisition in plants.

## EXPERIMENTAL PROCEDURES

### Plant materials

Arabidopsis ecotype Columbia (Col) was used as the WT control throughout this study. T‐DNA insertion lines for *HAK5* and *SKOR* (referred to as *hak5* (SALK_074868) and *skor* (SALK_132944), respectively) were obtained from the Arabidopsis Biological Research Center (ABRC). Note that the inserted T‐DNA was within the coding region (the fourth exon) of *HAK5* in the *hak5* mutant but within the first intron of *SKOR* in the *skor* mutant. Despite the position of the T‐DNA insertion site, the *SKOR* transcript level was remarkably reduced in the *skor* mutant (Alejandro et al., 2007). The *nlp7* mutant (*nlp7‐1*) was also obtained from the ABRC. The *nlp6nlp7* double mutant and *NLP7*‐OX transgenic line were generated previously (Liu et al., 2017; Maeda et al., 2018). The *NLP7*‐OX/*hak5* and *NLP7*‐OX*/skor* lines were generated by crossing the *NLP7*‐OX line with the *hak5* and *skor* mutants, respectively. Plants homozygous for both the *NLP7* transgene and either the *hak5* or *skor* mutant locus were selected through PCR‐based genotyping using PCR primers listed in Table S7.

### Plant growth conditions

Surface‐sterilized seeds were cold‐stratified at 4°C for 3 days and then sown on 1/2 MS agar plates (1/2 MS salts, 0.9% [w/v] agar, 0.5% [w/v] sucrose, and 3.4 mM MES‐KOH [pH 5.7]). The seedlings were grown in a growth chamber set at 23°C under continuous light at a light intensity of approximately 70 μmol m^−2^ s^−1^.

### Phenotypic analysis under K‐deficient conditions

To conduct phenotypic analysis on agar plates, seedlings grown for 5 days on 1/2 MS agar plates were transferred to vertically oriented agar plates for K‐sufficient (5 mM K^+^) or low K (2 μM K^+^) conditions and grown for 7 days. The agar plates used contained either 5 mM or 2 μM KCl, modified 1/2 MS salts (5.2 mM NH_4_NO_3_, 1.5 mM CaCl_2_, 0.75 mM MgSO_4_, 0.6 mM NaH_2_PO_4_, 0.05 mM H_3_BO_3_, 0.05 mM MnSO_4_, 0.015 mM ZnSO_4_, 0.5 μM Na_2_MoO_4_, 0.05 μM CuSO_4_, 0.05 μM CoCl_2_, 0.01 mM FeSO_4_, 0.05 mM Na_2_‐EDTA), 0.9% (w/v) agar, 0.5% (w/v) sucrose, and 3.4 mM MES‐NaOH (pH 5.7). Note that MES‐NaOH was used instead of MES‐KOH to adjust the pH, thereby allowing strict control of K^+^ concentration in the media.

We used lower nutrient concentrations compared with agar plate analysis for phenotypic analysis using a hydroponic system since nutrient diffusion is easier in liquid media and thus nutrient absorption is more effective. Seedlings were grown in a nutrient solution (modified 1/10‐strength MS salts solution) for 12 days and then transferred to K‐free nutrient solution with or without 1 mM KCl for 7 days in a growth chamber set at 23°C under continuous light.

### Measurement of ^86^Rb uptake activity with a radioisotope imaging system

WT and *nlp6nlp7* seedlings were grown on 1/2 MS medium for 6 days, followed by an additional 4 days on vertically oriented agar plates for low K (2 μM K^+^) conditions. After measurements of fresh weights, the roots were incubated in a K‐free 1/10 MS salts‐containing nutrient solution supplemented with ^86^RbCl (12 kBq ml^−1^) (POLATOM, Otwock, Poland) for 20 min. After washing the roots thoroughly with distilled water, images of ^86^Rb uptake were captured using an imaging plate (Fujifilm Co., Tokyo, Japan) and an FLA‐5000 fluorescent image analyzer (Fujifilm). To determine the uptake activities, the intensities of the ^86^Rb signals were quantified using ImageQuant TL software (GE HealthCare Japan, Tokyo, Japan) and a calibration curve prepared from a series of standard solutions, each diluted from the original ^86^Rb solution.

### RT‐qPCR analysis

To compare gene expression levels under K‐sufficient and low K conditions, seedlings were grown on 1/2 MS medium for 5 days, followed by an additional 4 days on vertically oriented agar plates for low K conditions (5 μM K^+^). To analyze nitrate‐induced gene expression, seedlings were grown in N‐free 1/10 MS liquid medium supplemented with 0.5 mM ammonium succinate for 5 days, and then treated with 10 mM KNO_3_ or KCl for 1, 3, or 6 h before harvest.

Total RNA was prepared from plant tissues with the Maxwell RSC Plant RNA Kit (Promega Co., Madison, WI, USA) following the manufacturer's protocol. For cDNA synthesis, total RNA (800 ng) and the oligo(dT)15 primer were used, together with Superscript II reverse transcriptase (Invitrogen, Thermo Fisher Scientific K.K., Tokyo, Japan). RT‐qPCR was performed with the synthesized cDNA, KAPA SYBR Fast qPCR mixture (KAPA Biosystems, MA, USA), and forward and reverse primers (Table S7) using the StepOnePlus system (Applied Biosystems, Thermo Fisher Scientific, Waltham, MA, USA). The relative expression levels of each gene were calculated using the 2^−ΔΔCT^ method (Livak & Schmittgen, 2001) and normalized against the expression level of *UBQ10* and then against the value obtained from the reference sample in each experiment.

### DNA microarray analysis

Total RNA was extracted from the roots of WT and *nlp6nlp7* seedlings that were grown for 5 days on 1/2 MS medium and then subjected to low K conditions (5 μM K^+^) for an additional 4 days. DNA microarray analysis was conducted according to the Agilent gene expression microarray one‐color protocol. First, cyanine 3‐labeled complementary RNA (cRNA) was synthesized from total RNA using the Low Input Quick Amp Labeling Kit, One‐color (Agilent Technologies, Santa Clara, CA, USA). The labeled cRNA was purified using the Qiagen RNeasy Mini Kit (Qiagen, Hilden, Germany) and subsequently hybridized with oligonucleotides on DNA microarrays (4 × 44 K format, Agilent Technologies) using the Gene Expression Hybridization Kit (Agilent Technologies). The DNA microarray slides were scanned using the DNA microarray scanner (Agilent DNA Microarray Scanner, G2565A, Agilent Technologies) and the Agilent Scan Control software ver. 7.0.03 (Agilent Technologies). The fluorescence signal intensity corresponding to each spot was evaluated using Feature Extraction Software Ver. 9.1 (Agilent Technologies). The mean values from three biological replicates were calculated to determine the expression levels for each gene. Student's *t*‐test was applied to identify significant differences in the expression levels of genes between seedlings cultivated under different K conditions, as well as between WT and *nlp6nlp7* seedlings. GO analysis was performed using The Gene Ontology Resource (https://geneontology.org). Heat maps were generated using the gplots package in the statistical analysis software R.

### Co‐transfection assay

The *35SΩ* promoter region in pJD301 plasmid (Luehrsen et al., 1992) was replaced with the *HAK5* promoter (−1611 to +77, relative to the transcription start site) or the *SKOR* promoter (−1602 to +85), generating two reporter plasmids containing the firefly *LUC*, pHAK5‐LUC, and pSKOR‐LUC. Another reporter plasmid containing *LUC* driven from the *NRT2.1* promoter, pNRT2.1‐LUC (Maeda et al., 2018), was used as the control. The effector plasmid, which expresses NLP7 under the control of a constitutive and strong promoter (pHBT‐NLP7‐MYC), the empty plasmid (pHBT‐none‐MYC) that does not contain any inserted cDNA between the promoter and the MYC epitope tag‐encoding sequence (Yanagisawa et al., 2003), and the UBQ10‐GUS internal control plasmid (Hwang & Sheen, 2001) have been described previously (Maeda et al., 2018).

Co‐transfection of the internal control plasmid, a reporter plasmid, and either pHBT‐NLP7 effector plasmid or the empty plasmid into mesophyll protoplasts was performed as described previously (Yoo et al., 2007). LUC and GUS activities in transfected protoplasts were measured, as described previously (Maeda et al., 2018).

### ChIP assay

ChIP assay was performed using *NLP7*‐OX seedlings grown on 1/2 MS medium for 10 days, as described previously (Ariga et al., 2022). qPCR using immunoprecipitated DNA was performed using pairs of PCR primers, which allowed amplification of different 200–300 bp regions of the *HAK5* and *SKOR* promoters (Table S7).

### EMSA

The recombinant protein used for EMSA was prepared from *Escherichia coli* (strain: BL21[DE3]) and the pET32‐NLP7DBD plasmid (Konishi & Yanagisawa, 2013), as described previously (Konishi & Yanagisawa, 2013). The pET32‐NLP7DBD plasmid directs the production of the DNA‐binding domain of NLP7 (amino acids 569–687), which is fused to a thioredoxin N‐terminus and a histidine (His) tag at both the N‐ and C‐termini. For preparation of DNA probes, specific region of *HAK5* (−43 to +80), *SKOR* (−1286 to −1164), and *NRT2.1* (−699 to −742) were PCR‐amplified using gene‐specific primers attached with a unique sequence for the annealing of biotin‐labeled primers (Table S7). Subsequently, a second PCR was performed with biotin‐labeled primers. The binding reaction of recombinant NLP7 to the DNA probes was carried out in a reaction mixture (20 mM HEPES‐KOH [pH7.6], 3 mM MgCl_2_, 30 mM KCl, 30 mM NaCl, 2 mM DTT, 0.5 mM EDTA, 10% [v/v] glycerol) at 23°C for 30 min. EMSA was performed, as described previously (Konishi & Yanagisawa, 2013). In competition assays, unlabeled DNA probes and synthetic DNAs generated by annealing two oligonucleotides (Table S7) were added to binding reaction mixtures as competitors at a 50‐fold molar excess compared with the labeled DNA probe.

## ACCESSION NUMBERS

Sequence data from this article can be found in the Arabidopsis Genome Initiative database under the following accession numbers: *UBQ10* (At4g05320), *NLP6* (At1g64530), *NLP7* (At4g24020), *HAK5* (At4g13420), *SKOR* (At3g02850), *NRT2.1* (At1g08090). Transcriptome data were deposited in the Gene Expression Omnibus (GEO) repository of the National Center for Biotechnology Information (NCBI) database under the accession number GSE327031 on April 11, 2026.

## AUTHOR CONTRIBUTIONS

SY conceived the research. KU, YS, and SY designed the experiments, and KU, MY, NO, NS, DT, and YS performed experiments. KU and SY wrote the article, along with input from all authors.

## CONFLICT OF INTEREST

The authors declare no conflict of interest.

## Supporting information


**Table S1.** Top 50 genes upregulated by cultivation under low K conditions in WT roots.

**Table S2.** Top 50 genes downregulated by cultivation under low K conditions in WT roots.

**Table S3.** Genes upregulated genes in *nlp6nlp7* roots under K‐sufficient conditions.

**Table S4.** Top 50 genes downregulated in *nlp6nlp7* roots under K‐sufficient conditions.

**Table S5.** Genes upregulated in *nlp6nlp7* roots under low K conditions.

**Table S6.** Genes downregulated in *nlp6nlp7* roots under low K conditions.

**Table S7.** List of oligonucleotides used in this study.

## Data Availability

The data that support the findings of this study are available on request from the corresponding author. The data are not publicly available due to privacy or ethical restrictions.
